# Research Hotspots and Emerging Frontiers in Ovarian Aging: A Bibliometric Analysis (2006–2025)

**DOI:** 10.2174/0118715303441084260119050045

**Published:** 2026-04-28

**Authors:** Qingquan Gong, Wenhong Ma, Chao Yang, Dingyu Liang, Yifu Wang, Yihua Yang, Mingyou Dong

**Affiliations:** 1 Guangxi Reproductive Medical Center, the First Affiliated Hospital of Guangxi Medical University, Nanning, 530021 China;; 2 Key Laboratory of Early Prevention and Treatment for Regional High Frequency Tumor (Guangxi Medical University), Ministry of Education, Nanning, 530021, China;; 3 Reproductive Medicine Center, Nanning Women and Children's Hospital, Nanning, 530011, China;; 4 Guangxi Engineering Research Center for Precise Genetic Testing of Long-dwelling Nationalities, Youjiang Medical University for Nationalities, Baise, 533000, China

**Keywords:** Ovarian Aging, bibliometric analysis, mitochondrial dysfunction, cellular senescence, inflammation, fibrosis, multi-omics

## Abstract

**Introduction:**

Ovarian aging has a critical impact on women's fertility and overall health. This study uses bibliometric methods to analyze the current state of research in the field of natural ovarian aging and to identify emerging trends.

**Methods:**

This study analyzed 653 publications from the Web of Science Core Collection (2006-2025). Using VOSviewer and CiteSpace software, the authors visualized collaboration networks and identified research hotspots through co-occurrence and co-citation analyses.

**Results:**

Annual publications in the field of natural ovarian aging showed a significant upward trend; China and the United States were the main contributors to this research area. Huazhong University of Science and Technology and Nanjing Medical University were among the institutions with the highest number of publications. Wang SX was the author with the most publications, while Broekmans FJ was the most cited scholar. Human Reproduction was a core journal in this field. Keyword co-occurrence and reference co-citation analyses indicated that mitochondrial dysfunction, cellular senescence, inflammation, fibrosis, and multi-omics research were the research hotspots and frontiers during the study period.

**Discussion:**

The surge in keywords and citations suggests that the field is undergoing a fundamental shift from clinical phenotype studies to the exploration of molecular mechanisms. Findings in hotspot areas such as fibrosis and senescent cell clearance provide potential therapeutic targets for future clinical translation.

**Conclusion:**

This study employs bibliometric methods to map the current state of research, core themes, and emerging frontiers in the field of natural ovarian aging. The results offer researchers valuable insights into understanding the development trends in this field and guiding future research.

## 1 INTRODUCTION

Ovarian aging is a complex physiological process driven by multiple factors, characterized by a gradual decline in ovarian follicle reserve and oocyte quality. These changes ultimately result in the loss of reproductive capacity and decreased endocrine function [1-3]. Ovarian aging directly affects female fertility and increases the risk of infertility, miscarriage, and birth defects [4-7]. It is also closely associated with the occurrence of age-related diseases such as cardiovascular disease, osteoporosis, and cognitive decline, thereby impacting women's quality of life and life expectancy [8, 9].

Importantly, although Premature Ovarian Insufficiency (POI) shares similar clinical features with natural aging, it is a pathological condition with causes and progression distinct from natural aging [10]. Natural ovarian aging is an inevitable physiological stage with broader public health implications [11, 12]. Therefore, this study specifically focuses on the mechanisms and trends associated with the natural aging process.

Bibliometrics is an interdisciplinary field that uses mathematical and statistical methods to analyze publication data quantitatively [13]. By visualizing data such as countries, institutions, authors, journals, keywords, and citations, this approach can effectively map the knowledge structure, development, research hotspots, and future trends within a specific field [14]. Bibliometrics has been widely applied in many medical fields, including obstetrics and gynecology [15, 16], oncology [17], and cardiovascular research [18].

However, there is currently a lack of comprehensive bibliometric analysis in the research field of ovarian aging. This study utilizes bibliometric methods to analyze the annual publication trends in ovarian aging research and identify key countries, institutions, journals, and authors. Additionally, the study delves into co-cited references, high-frequency keywords, and emerging terms to outline the knowledge structure, research hotspots, and frontier dynamics in natural ovarian aging research (*e.g.*, inflammation, fibrosis, mitochondrial dysfunction, cellular senescence, multi-omics studies). These findings will help researchers gain a comprehensive understanding of ovarian aging research and provide valuable references for future research directions.

## 2 METHODS

### 2.1 Data Source and Search Strategy

This study selected the Web of Science Core Collection (WoSCC) as the data source because it is widely regarded as an authoritative database of high-quality academic literature and provides comprehensive citation metadata. These data are essential for the precise co-citation analysis conducted in this study. The retrieval time range was from January 1, 2006, to May 1, 2025. A nearly twenty-year time span was chosen to capture the dynamic evolution of the field, particularly the paradigm shift from descriptive studies to mechanism exploration driven by high-throughput technologies. The search query was as follows: TI=(ovary OR ovarian) AND TI=(aging OR senescence). The search results were then filtered by limiting the language to English and restricting the document types to “articles” and “reviews,” ultimately yielding 1,380 publications.

Subsequently, two authors independently screened these publications manually, excluding studies that did not directly focus on ovarian aging, such as those primarily concerning polycystic ovary syndrome or ovarian cancer. To ensure reliability, any disagreements regarding the inclusion of literature were first discussed between the two reviewers. If consensus could not be reached, a third senior researcher was consulted to make the final decision. After this process, the final dataset included 653 publications. For each included publication, complete metadata (including title, authors, institutions, abstract, keywords, and references) were downloaded and saved as plain text files, which were then imported into bibliometric analysis software.

### 2.2 Bibliometric Analysis

This study used two main bibliometric software tools for data analysis and visualization: VOSviewer (version 1.6.20) and CiteSpace (version 6.4.R2).

VOSviewer was used to construct and visualize scientific knowledge maps [19]. This included collaboration network analysis of countries, authors, institutions, and journals, as well as co-occurrence network analysis of keywords. These network maps illustrated the strength of relationships and clustering patterns among the analyzed entities.

CiteSpace was used to identify research hotspots and emerging trends [20]. It performed co-citation analysis to generate cluster maps and timeline views, which helped identify core literature clusters and their evolution over time. Additionally, CiteSpace was used to detect keyword bursts and pinpoint significant research topics and emerging trends. The main parameter settings were as follows: time slicing: 2006-2025 (2-year slices); selection criteria: g-index (k=25), LRF=3.0, L/N=10, LBY=5, e=1.0; pruning: Pathfinder.

Fig. (**1**) shows a detailed flowchart of data collection and processing.

## 3 RESULTS

### 3.1 Analysis of Overall Literature Characteristics and Publication Trends

By searching the WoSCC database, we found 653 publications on ovarian aging, including 564 original articles (86.4%) and 89 reviews (13.6%). These publications were written by 3,474 researchers from 897 institutions in 53 countries, published in 251 different journals, and cited a total of 25,554 references.

**Fig. (2):**
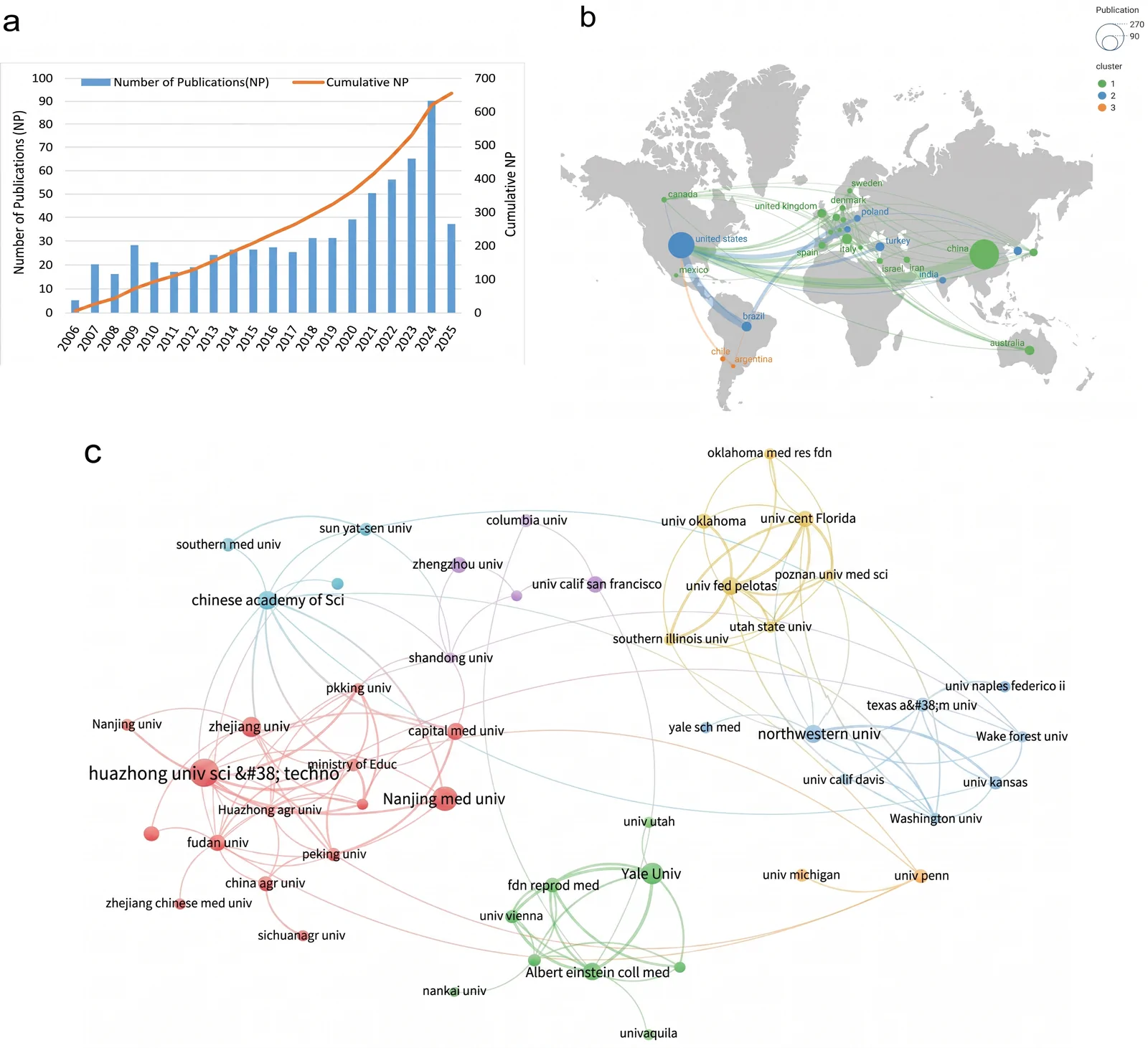
Publication trends and collaboration networks in ovarian aging research. (**a**) Annual and cumulative publications (2006–2025). (**b**) The global distribution and international collaboration network of publications. (**c**) The co-authorship network of research institutions.

The annual number of publications in this field showed a general upward trend with some fluctuations (Fig. **2a**). This growth accelerated significantly after 2019, reaching a peak of 90 articles in 2024, the highest volume in the last two decades. The cumulative publication curve showed a continuous, steep rise, exceeding 650 articles by May 2025. This indicates that ovarian aging is currently a growing area of academic interest and is in a phase of rapid development.

### 3.2 Analysis of Countries, Institutions, and Their Collaboration Networks

As shown in Table **1**, China was the most productive country in ovarian aging research, with 261 publications (39.97%). The United States followed closely with 199 publications (30.47%). Other countries with a high number of publications included Italy (29 articles, 4.44%), Brazil (28 articles, 4.29%), and Australia (26 articles, 3.98%).

Fig. (**2b**) shows the collaboration network between countries. In the network map, the size of a node represented the number of published papers, and the links between nodes represented collaborative relationships. China and the United States were the two largest nodes, indicating that both countries had high paper output and extensive collaboration with other countries. Other countries, such as the United Kingdom, Canada, Australia, and Italy, were also actively involved in international cooperation, forming several distinct subnetworks. Overall, international collaboration in this field was active, forming a network centered around China and the United States.

At the institutional level, Huazhong University of Science and Technology ranked first with 33 papers (Table **1**). Nanjing Medical University (25 papers) and Yale University (19 papers) ranked second and third, respectively. Among the top ten institutions by paper output, six were from China, and three were from the United States, further highlighting the significant contributions of these two countries. Fig. (**2c**) showed the institutional collaboration network. A prominent collaboration cluster (highlighted in red) was observed among Chinese institutions, including Huazhong University of Science and Technology, Nanjing Medical University, and Capital Medical University.

### 3.3 Analysis of Journals and Co-cited Journals

Table **2** lists the 10 journals with the highest number of published papers in ovarian aging research. The three journals with the most publications were Human Reproduction (25 articles), Aging-US (24 articles), and Fertility and Sterility (24 articles). Most of these journals were in the Q1 or Q2 quartiles of the Journal Impact Factor (JIF) (except for Aging-US), indicating that research in this field was generally published in high-impact journals.

In co-citation analysis, Human Reproduction also ranked first with 1,948 co-citations. Fertility and Sterility (1,841 co-citations) and Biology of Reproduction (1,007 co-citations) ranked second and third, respectively. These journals were considered highly influential in the fields of obstetrics, gynecology, and reproductive medicine.

### 3.4 Analysis of Authors and Their Collaboration Networks

Table **3** highlights the main contributors in this field. Papers by Wang SX (22 articles) and Wu M (15 articles) ranked at the top in terms of publication volume, indicating their key role in driving recent research outputs. However, in terms of academic influence, Broekmans FJ (208 citations) and Faddy MJ (167 citations) had the highest co-citation counts. This comparison suggested that although new research teams (such as Wang's team) were accelerating knowledge production, the field still largely relied on the conceptual frameworks established by these highly cited scholars.

**Fig. (3):**
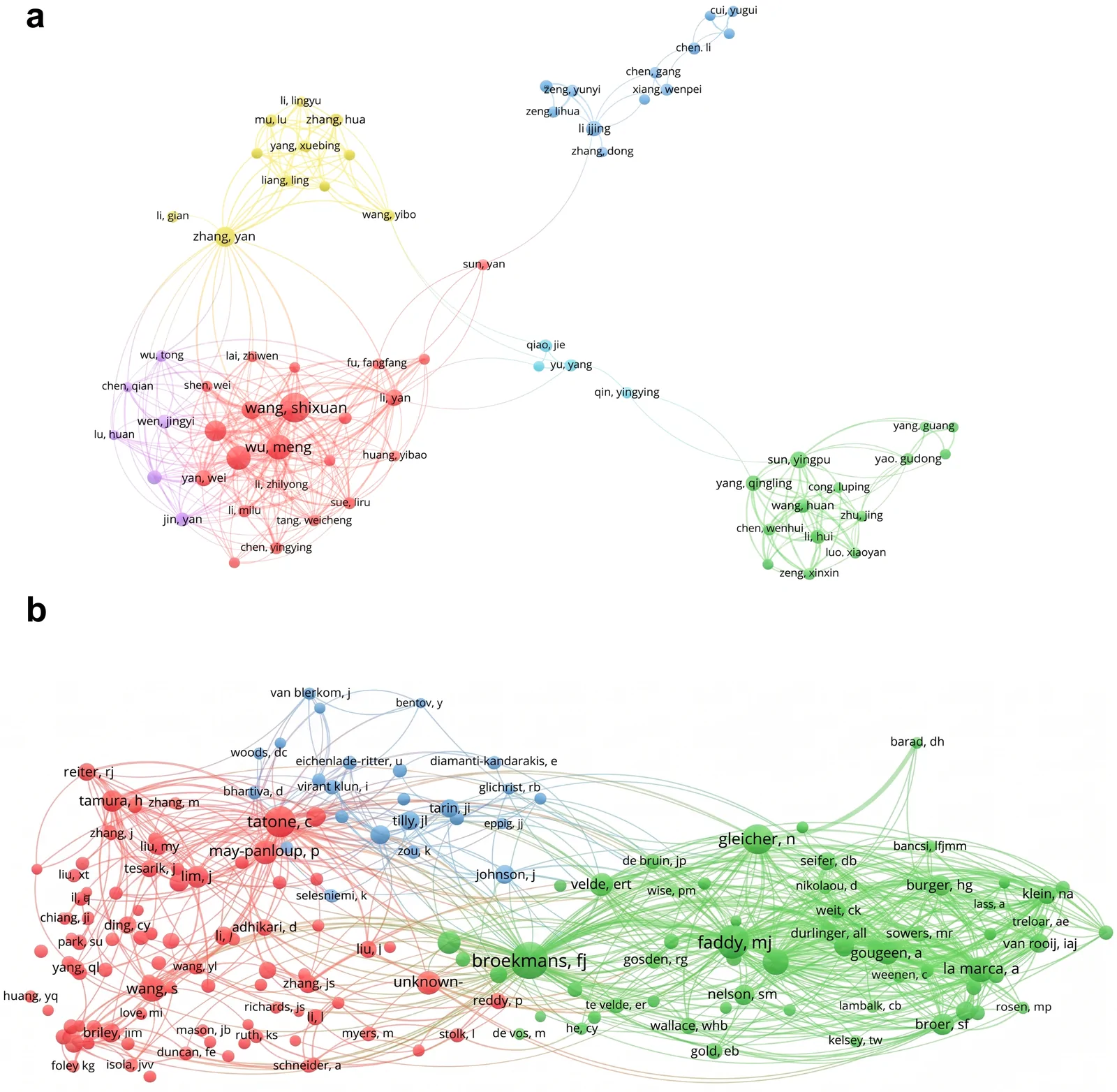
Author collaboration and co-citation networks in ovarian aging research. (**a**) The author collaboration network. (**b**) The co-cited author network.

In addition to individual rankings, Fig. (**3**) illustrates the social structure of the research community. The collaboration network (Fig. **3a**) revealed several distinct, closely connected clusters, which mainly formed around high-output research teams (such as the team led by Wang SX). These clusters indicated that the research efforts were often organized within specific institutional or regional groups. In contrast, the co-citation network (Fig. **3b**) demonstrated a more integrated structure with highly connected central nodes. Scholars such as Broekmans FJ and Tatone C occupied these central positions, linking diverse research foci and serving as the intellectual “bridges” that connected different sub-fields of ovarian aging research.

### 3.5 Identifying Knowledge Structure and Frontier Evolution through Co-citation Analysis of Literature

Table **4** lists the 10 most co-cited publications (the complete list can be found in Supplementary Table **S1**). Wang S's “Single-Cell Transcriptomic Atlas of Primate Ovarian Aging,” published in Cell in 2020, ranked first with 72 co-citations [21]. Park SU's review on ovarian aging mechanisms [1] and Lliberos C's study on inflammation and follicular depletion assessment during mouse ovarian aging [22] also had high co-citation frequencies of 35 and 32, respectively. These highly co-cited articles, which primarily explore mechanisms such as inflammation, oxidative stress, and mitochondrial dysfunction, represent foundational research in the field.

Citation burst analysis identified publications that experienced a surge in citations over a specific period, typically revealing the field's research frontiers at that time. In this study, we identified the top 25 references with the highest burst strength (see Supplementary Table **S2** for details). Among them, the article by May-Panloup P. published in 2016, “Ovarian Aging: The Role of Mitochondria in Oocytes and Follicles” [23], showed the most significant burst in citations (strength = 12.25). This finding indicated that research on the mechanisms of ovarian aging had shifted its focus toward mitochondrial dysfunction.

**Fig. (4):**
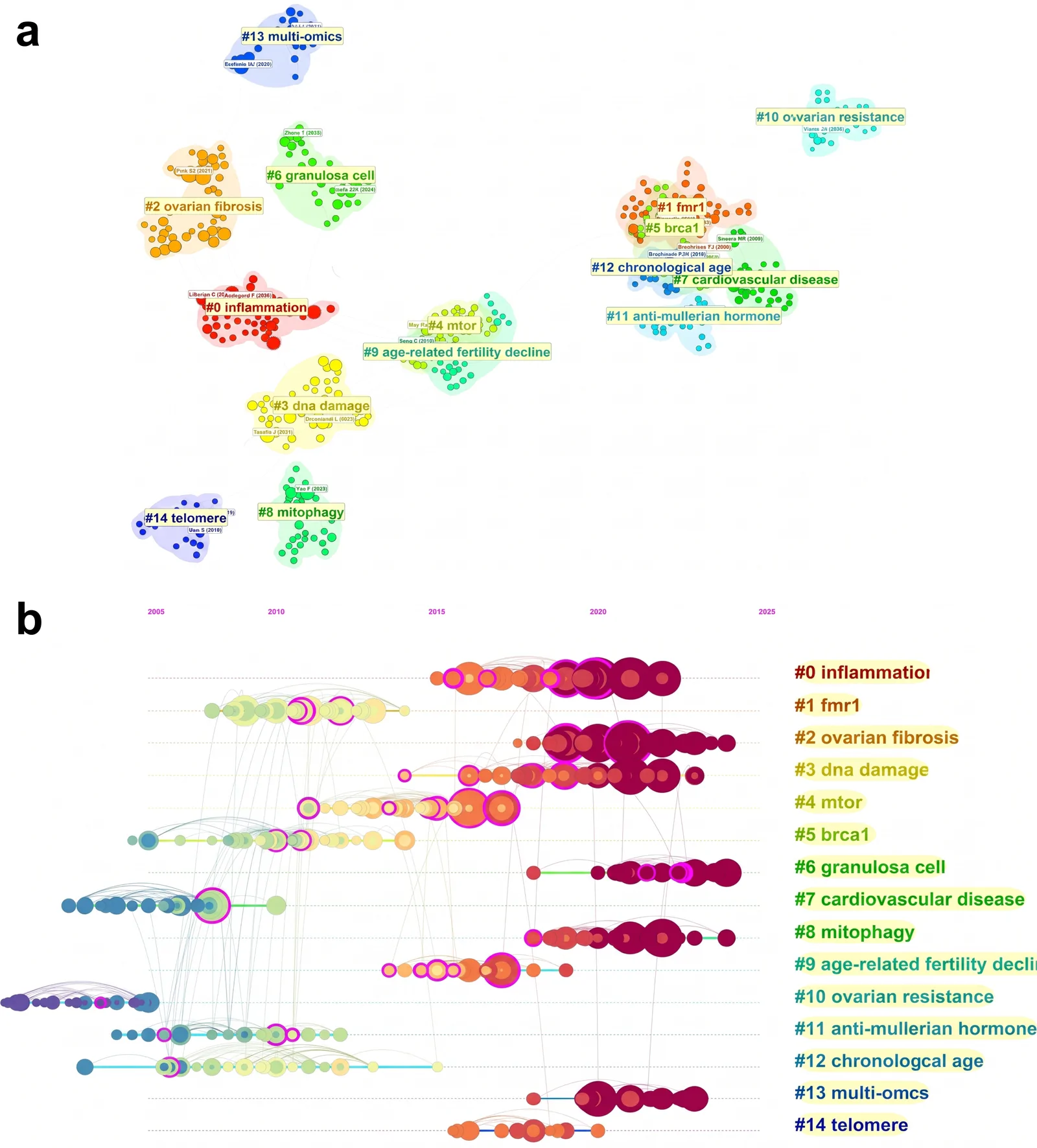
Cluster map and timeline view of the reference co-citation network in ovarian aging research. (**a**) Cluster map of the reference co-citation network. (**b**) Timeline view of co-citation clusters.

Through clustering analysis of co-cited literature (Fig. **4a**), both the modularity (Modularity Q = 0.88) and the mean silhouette score (Mean Silhouette S = 0.96) were high, indicating that the clustering was effective and the structure was clear. A total of 15 major clusters were formed, representing the core topics and directions in ovarian aging research. Among them, the larger clusters included: #0 Inflammation, #2 Ovarian Fibrosis, and #3 DNA Damage, among others.

The Timeline View in Fig. (**4b**) illustrates the evolution of these research themes over time. In the earlier period (around 2006–2015), research focused on topics such as “fmr1” (#1), “brca1” (#5), and “anti-Müllerian hormone” (#11). In recent years (after 2018), there was a significant increase in publications within clusters such as #0 inflammation, #2 ovarian fibrosis, #3 DNA damage, #8 mitophagy, and #13 multi-omics. This suggested these topics were current research hotspots and frontiers. The “inflammation” cluster (#0) in particular showed high, sustained activity over the past five years (2020–2025), making it one of the most prominent current research frontiers.

### 3.6 Mapping Research Hotspots and Development Trends through Keyword Analysis

**Fig. (5):**
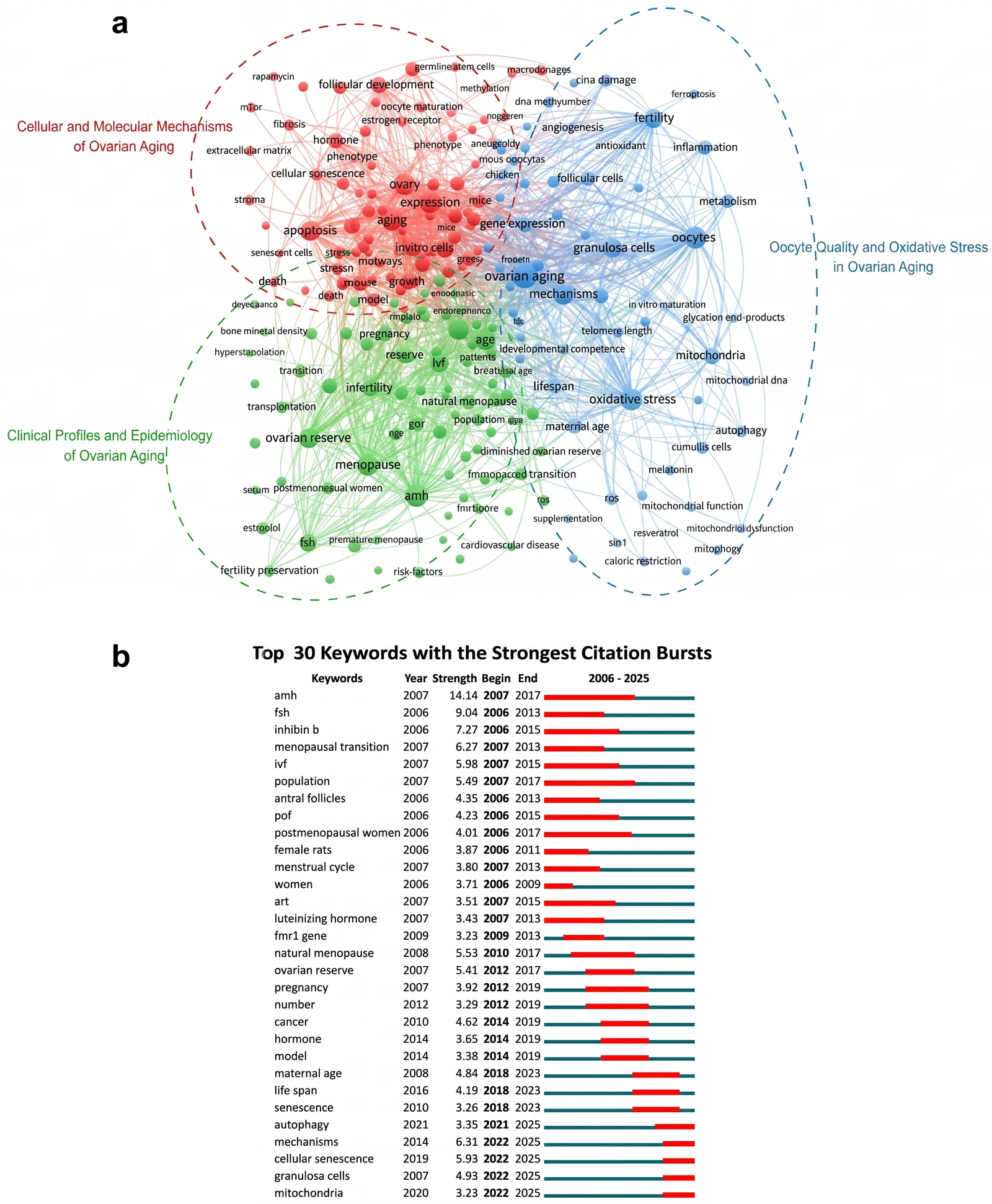
Analysis of research hotspots and frontiers through keyword co-occurrence and burst detection. (**a**) The keyword co-occurrence network reveals three main research domains. (**b**) The top 30 keywords with the strongest citation bursts.

The keyword co-occurrence network (Fig. **5a**) illustrated the core topics in ovarian aging research and their connections. High-frequency keywords include oocytes, fertility, oxidative stress, mitochondria, inflammation, apoptosis, menopause, ovarian reserve, and AMH(anti-Müllerian hormone). The network formed three main clusters.

The red cluster focused on the cellular and molecular mechanisms of ovarian aging. This included research on: (1) Physiological and pathological processes of ovarian structural and functional changes with age, such as follicular atresia, stromal fibrosis, and altered angiogenesis; (2) Research content broadly covered the roles of gene expression (gene, expression, messenger-RNA, microRNA), proteins, and key signaling pathways such as the mTOR pathway (mTOR, rapamycin) in ovarian aging; (3) Exploration of the potential of stem cells (stem cells, germ cells, germline stem cells, bone marrow, c-kit) in maintaining and repairing ovarian function.

The green cluster primarily focused on the clinical aspects of ovarian aging and its impact on reproductive health. The key topics of this cluster included: (1) clinical diagnostic markers, such as AMH, FSH, inhibin B, and estradiol; (2) female fertility, including assisted reproductive technologies (ART, IVF, pregnancy rate, live birth rate) and fertility preservation; (3) the systemic effects of ovarian aging on overall female health, such as cardiovascular diseases (cardiovascular disease, coronary heart disease), osteoporosis (bone density), metabolic syndrome (metabolic syndrome, obesity, blood lipids, insulin resistance), and mood disorders (depression). This cluster emphasized clinical attention to the diagnosis and management of ovarian aging and its broader implications for lifelong female health.

The blue cluster focused on the molecular mechanisms of oocyte aging and potential interventions. This research involved: (1) the roles of mitochondria (mitochondria, mitochondrial DNA, mitochondrial dysfunction, mitophagy) and oxidative stress (oxidative stress, antioxidants) in oocytes; (2) regulatory pathways, such as the Sirtuins pathway (SIRT1, sirtuins, NAD(+)); (3) potential therapeutic strategies, including antioxidants (antioxidants, melatonin, resveratrol), calorie restriction, and mesenchymal stem cell therapy, aimed at improving oocyte quality to delay ovarian aging.

Finally, keyword burst analysis identified 30 keywords with relatively high burst strength, revealing changes in research frontiers over time (Fig. **5b**). In the early period (2006-2017), keywords with high burst strength included “AMH,” “FSH,” “inhibin B,” “IVF,” “POF,” and “perimenopause.” These keywords were mainly related to the clinical assessment of ovarian function and associated diseases. In recent years, keywords extending to the period 2021-2025 included “autophagy,” “mechanism,” “cellular senescence,” “granulosa cells,” and “mitochondria.” The emergence of these keywords indicated a shift in research frontiers towards the potential cellular and molecular mechanisms of ovarian aging, with particular attention to processes such as autophagy and the roles of key cells and organelles.

## 4 DISCUSSION

### 4.1 General Information

This study uses bibliometric methods to conduct a systematic visual analysis of literature related to ovarian aging from 2006 to 2025, aiming to identify the main contributors, research hotspots, and development trends in this field. The results indicate that the field of ovarian aging is developing rapidly, with research focus shifting from macroscopic clinical manifestations to microscopic molecular mechanisms.

In this study, China and the United States are the main contributors to research output, accounting for the vast majority of published papers. This may be attributed to substantial investment in scientific research, well-established research infrastructure, and the high emphasis on reproductive and geriatric medicine in both countries.

Institutions such as Huazhong University of Science and Technology, Nanjing Medical University, and Yale University have been highly productive and are influential research centers in this field. An analysis of the collaboration network shows that an international network centered around the United States has formed. At the same time, collaboration among Chinese institutions and between Chinese and international institutions has also grown stronger. This trend facilitates knowledge dissemination and enhances the overall research quality. However, the international collaborations of Chinese institutions are less extensive than those involving the US. Future efforts should focus on promoting more substantive international collaborative projects.

As the most prolific author in the field of natural ovarian aging, Wang SX and his team have made systematic and multidimensional contributions. His research has explored the molecular mechanisms of ovarian aging (*e.g.*, Sirtuins, WIP1, FOXP1) [24-26]. Moreover, Wang provided the first spatial transcriptomics map of human ovarian aging, offering new insights into cellular heterogeneity and microenvironmental interactions [26]. Furthermore, his team has actively explored various potential intervention strategies, including the protective effects of resveratrol [27], metformin [28], and Kuntai Capsule [29], as well as the use of SIRT1/TGFBR2 gene therapy to delay ovarian aging [24]. Finally, the team's reviews on the hallmarks of aging [30], the stromal microenvironment [31], and pharmacological strategies [32] have provided a theoretical foundation for understanding mechanisms, developing interventions, and guiding clinical practice.

The top 10 most co-cited publications reflect the key scientific questions and advances in ovarian aging research. Ranking first is “Single-Cell Transcriptomic Atlas of Primate Ovarian Aging,” published in Cell [21]. This study used single-cell transcriptomics to map the transcriptional landscape of primate ovarian aging, offering a new perspective for understanding cellular heterogeneity and dynamic gene expression changes at the single-cell level. This paper demonstrates the power of single-cell technologies for studying complex biological processes and marks the entry of ovarian aging research into the single-cell era. This finding aligns with the research front “#13 multi-omics” identified in our co-citation cluster analysis. Other highly co-cited articles focus on themes such as the mechanisms of ovarian aging [1], inflammation [22, 33], ovarian stromal stiffening [34], oxidative stress [35, 36], and mitochondrial function [23, 37, 38].

Keywords, as a condensation of a paper's core content, can be used in a co-occurrence network to identify a field's research priorities effectively. The keyword co-occurrence network reveals three major research domains in ovarian aging: 1. Cellular and molecular mechanisms of ovarian aging (*e.g.*, apoptosis, cellular senescence, gene expression, shown in the red cluster); 2. Clinical manifestations and assessment of ovarian aging (*e.g.*, ovarian reserve, menopause, AMH, IVF, cardiovascular disease, shown in the green cluster); and 3. Oocyte quality and improvement strategies in ovarian aging (*e.g.*, oocytes, oxidative stress, antioxidants, mitochondria, DNA damage, sirtuins, shown in the blue cluster).

### 4.2 Research Hotspots and Frontiers

By integrating keyword burst analysis and literature co-cited cluster analysis, this study identifies the following four current research hotspots and frontiers in natural ovarian aging (A schematic diagram is shown in Fig. (**6**).

#### 4.2.1 Mitochondrial Homeostasis and Ovarian Aging

As the “powerhouses” of the cell, the functional state of mitochondria is crucial for oocyte quality and overall ovarian function [39, 40].

##### 4.2.1.1 Reactive Oxygen Species (ROS) and Oxidative Stress

Mitochondria are the primary source of intracellular ROS. Studies have shown that, with aging, the antioxidant capacity of ovarian tissue declines, and inefficient ROS clearance exacerbates oxidative stress [41]. Excessive ROS can damage mitochondrial structure and function, accelerating oocyte apoptosis and follicular atresia [42].

##### 4.2.1.2 Mitochondrial DNA (mtDNA) Damage

Due to the lack of effective histone protection and robust repair mechanisms, mtDNA is highly susceptible to attacks by ROS, leading to mutations and deletions [43]. This, in turn, impairs mitochondrial function and is considered an early event in ovarian aging [44, 45].

##### 4.2.1.3 Mitophagy

As a selective cellular quality control mechanism for clearing damaged mitochondria, the efficiency of mitophagy may decline during ovarian aging [46]. This leads to the accumulation of dysfunctional mitochondria, further exacerbating oxidative stress and cellular dysfunction [47].

##### 4.2.1.4 NAD+ (Nicotinamide Adenine Dinucleotide)

NAD+ is an important coenzyme in cellular energy metabolism and signal transduction, and its levels decrease significantly with age [48, 49]. NAD+ is also an essential co-substrate for the activity of the Sirtuin protein family, which plays a central role in maintaining mitochondrial homeostasis [50, 51]. Therefore, age-related declines in NAD+ levels reduce Sirtuin activity, impair mitochondrial oxidative phosphorylation, and increase oxidative stress. These changes collectively promote mitochondrial dysfunction, accelerate oocyte quality decline, and deplete ovarian reserves [52, 53]. Thus, supplementation with NAD+ precursors or activation of Sirtuins has become a research focus for improving mitochondrial function and delaying ovarian aging [54].

Targeting mitochondrial quality control pathways provides a direct therapeutic approach to restoring oocyte function and extending reproductive longevity.

#### 4.2.2 Cellular Senescence and Ovarian Function Decline

Cellular senescence is a state of permanent cell cycle arrest induced by natural aging or stress, accompanied by a series of distinct phenotypic changes. Persistent DNA damage is a major driver of cellular senescence [55-57]. Ovarian cells are susceptible to DNA damage caused by both endogenous and exogenous factors, thereby activating the DNA Damage Response (DDR) [58]. If the damage cannot be repaired, it leads to cellular senescence, characterized by increased activity of senescence-associated β-galactosidase (SA-β-Gal) and upregulation of cell cycle regulators p16 and p21 [59]. Additionally, senescent cells secrete inflammatory cytokines and chemokines, forming a Senescence-Associated Secretory Phenotype (SASP). This secretion alters the ovarian microenvironment and exacerbates tissue damage, ultimately promoting ovarian aging [60].

Wu and colleagues mapped the spatiotemporal transcriptomic landscape of human ovarian aging and confirmed that cellular senescence is a common phenotype throughout the aging process. They also found that FOXP1 expression in the ovary decreases significantly with age. FOXP1 can directly bind to the p21 promoter and inhibit its transcription. Therefore, the downregulation of FOXP1 in aging ovaries removes its inhibitory effect on p21, thereby promoting the progression of cellular senescence [26].

Drugs that selectively eliminate senescent cells—senolytics—are currently a hotspot in anti-aging research [61, 62]. Wu and colleagues also found that quercetin, a senolytic, can reduce cellular senescence and effectively protect ovarian reserve in middle-aged mice [26]. Exploring the application of senolytics in ovarian aging represents a promising intervention strategy.

Using senolytics to selectively remove senescent cells provides a novel and translationally valuable approach to delaying ovarian aging and extending reproductive lifespan.

#### 4.2.3 Ovarian Microenvironment Remodeling and the Aging Process

The ovarian microenvironment supports follicle growth and development. It is a key component of ovarian aging, and its dysfunction is an important feature of the ovarian aging process.

##### 4.2.3.1 Inflammation

Chronic low-grade inflammation is a common hallmark of aging (also known as “inflammaging”) [63, 64]. During ovarian aging, factors such as SASP and oxidative stress increase the release of pro-inflammatory cytokines and alter immune cell infiltration [33, 65]. This creates a local inflammatory microenvironment in the ovaries, accelerating follicular atresia and interstitial fibrosis. Recent studies provide direct evidence for this theory. Lliberos *et al*. found that during natural aging in mice, the expression levels of pro-inflammatory cytokines (*e.g.*, TNF-α, IL-6, IL-1β) and key inflammasome components (*e.g.*, NLRP3, ASC) were significantly upregulated in ovarian tissue as mice aged. In addition, the composition of immune cells in senescent ovaries is altered, with a significant increase in macrophages, B cells, and CD4+ T cells, which contribute to the formation of local inflammatory states [22].

Furthermore, the study by Navarro-Pando *et al*. not only explores this phenomenon but also suggests potential intervention strategies [66]. They observed significant upregulation of key components of the NLRP3 inflammasome (NLRP3, caspase-1, IL-1β). This upregulation is present in both the ovaries of aging mice and in granulosa cells of patients with reduced ovarian reserve. Inhibition of NLRP3 function by gene knockout (Nlrp3−/−) or specific inhibitor (MCC950) can effectively delay ovarian aging, improve ovarian reserve function, restore ovulation function, and increase pregnancy rate. This provides strong evidence that targeting this inflammasome is a promising therapeutic strategy for delaying ovarian aging.

##### 4.2.3.2 Ovarian Fibrosis

Fibrosis is another key pathological feature of ovarian aging [67], characterized by excessive deposition of extracellular matrix and increased tissue hardness [68, 69]. This process can restrict follicle growth and hinder ovulation, ultimately leading to ovarian failure [70, 71]. Recent studies have made some progress in developing intervention strategies for ovarian fibrosis.

A study by Umehara *et al*. revealed for the first time that mitochondrial dysfunction in ovarian stromal cells can lead to fibrosis and showed that this process is reversible [72]. They demonstrated that treatment with anti-fibrotic drugs such as pirfenidone and BGP-15 can effectively reverse ovarian fibrosis that has formed in an elderly or obese mouse model. The underlying mechanism involves improving mitochondrial function and upregulating matrix metalloproteinase 13 (MMP13) expression. This upregulation promotes the degradation and removal of excess collagen. Ultimately, these changes restored ovulatory function and extended the female mouse's reproductive lifespan. In another study, Landry and colleagues identified a potential mechanism for preventing ovarian fibrosis through immune regulation [73]. Using single-cell sequencing, they found that although metformin cannot treat established fibrosis, it can prevent its development by remodeling the ovarian immune microenvironment. Specifically, metformin promotes the formation of regulatory macrophage subsets. These macrophages effectively suppress the accumulation of pro-inflammatory, SASP-positive fibroblasts, thereby preventing fibrosis.

Reversing ovarian stromal fibrosis and eliminating chronic inflammation are necessary prerequisites for restoring a microenvironment conducive to follicle development.

#### 4.2.4 Integrated Application of Multi-Omics Technologies

With the advancement of high-throughput sequencing technology, multi-omics research has provided unprecedented opportunities to reveal the complex molecular mechanisms of ovarian aging [74].

##### 4.2.4.1 Transcriptomics (Bulk Sequencing + Single-Cell Sequencing)

By analyzing ovarian tissue using bulk RNA sequencing (Bulk RNA-seq) or single-cell RNA sequencing (scRNA-seq), key genes and signaling pathways related to ovarian aging can be identified [75-77]. Notably, scRNA-seq can reveal the heterogeneity of different cell types within the ovary (such as oocytes [21], granulosa cells [26], stromal cells [26, 78], and immune cells [78]) and their dynamic changes during aging at the single-cell level.

In the follicles—which constitute the core functional units of the ovary—the study by Wang *et al*. provided key insights [21]. They conducted single-cell sequencing of primate ovaries to construct a detailed aging map. They noted that early oocytes and granulosa cells are the primary targets of oxidative stress-induced damage during aging. In addition, they found that gene expression in antioxidant pathways in these cells significantly decreases with age, providing direct molecular evidence for the decline in oocyte quality and granulosa cell dysfunction.

At the level of the stromal microenvironment that supports follicle development, the study by Zhou *et al*. focused on stromal cells and immune cells [78]. Through single-cell analysis of human ovaries, they found that pyroptotic macrophages accumulate with age, shifting the immune microenvironment from a 'regulatory' to a 'pro-inflammatory' state. This immune-driven remodeling accelerates stromal cell aging and fibrosis, ultimately leading to the decline of ovarian function.

##### 4.2.4.2 Genomics

Identifying gene variants associated with ovarian aging through techniques such as Genome-Wide Association Studies (GWAS) is crucial for understanding genetic susceptibility. Natural age at menopause (ANM) is commonly used as a core phenotype [79].

A landmark GWAS study conducted by Ruth and colleagues involving approximately 200,000 women increased the number of known ANM-associated genetic loci from 56 to 290 [80]. The study also revealed that DNA Damage Repair (DDR) pathways regulate the establishment and depletion of ovarian reserve. Subsequent interventions targeting key DDR genes in mice, such as CHEK1 and CHEK2, extended their reproductive lifespan. Furthermore, the study used Mendelian randomization to show that a longer reproductive lifespan has broader health impacts, including improved bone health and decreased risk of type 2 diabetes; however, it may increase the risk of hormone-sensitive cancers.

A follow-up study by Stankovic and colleagues focused on rarer protein-coding variants with larger effects. By analyzing exome data from over 100,000 women, they identified several novel genes significantly associated with ANM, such as SAMHD1 and ZNF518A [81]. The effect sizes of rare variants in these genes were approximately five times greater than those of common variants. This work strengthened the genetic link between ovarian aging and cancer risk (for example, SAMHD1 variants extend female reproductive lifespan but increase overall cancer risk). It also suggests that offspring of mothers with a genetic risk for ovarian aging may have higher de novo mutation rates, offering a new perspective on the intergenerational impact of reproductive aging.

##### 4.2.4.3 Epigenetics

Studies have shown that epigenetic modifications, such as DNA methylation and chromatin accessibility, play key regulatory roles in ovarian aging.

For instance, at the oocyte level, Potabattula and colleagues’ research on human and mouse oocytes found that methylation levels in the ribosomal DNA (rDNA) promoter region significantly increase with maternal age [82]. This age-dependent high rDNA methylation may affect ribosome biogenesis, which is crucial for oocyte growth and subsequent embryo development.

From a broader perspective, Jin and colleagues constructed the first single-cell multi-omics atlas of human ovarian aging [83]. Their study showed that the transcriptome and chromatin accessibility of multiple ovarian cell types undergo coordinated changes during aging. The study identified ovarian-specific aging pathways, such as the mTOR signaling pathway. It also integrated GWAS data, linking genetic risk loci associated with ovarian dysfunction to gene regulatory networks in specific cell types, providing molecular evidence of how genetic factors influence ovarian function through epigenetic modifications.

##### 4.2.4.4 Proteomics and Metabolomics

Analyzing protein and metabolite profiles of ovarian tissue or follicular fluid can help discover new biomarkers and understand metabolic reprogramming during ovarian aging.

A proteomics study revealed the presence of many “extra-Long-Lived Proteins” (LLPs) in mammalian ovaries [84]. These proteins have extremely long half-lives and are essential for maintaining mitochondrial function, chromatin structure, and protein homeostasis. However, the study also found that the abundance of these protective LLPs, particularly those involved in maintaining protein homeostasis, decreased significantly with ovarian aging. The deletion of this key protein is thought to be a potential mechanism leading to decreased ovarian function and fertility.

In metabolomics, a recent study comparing follicular fluid across age groups of women undergoing assisted reproductive technology showed significantly higher levels of various amino acids and lipid-related metabolites (*e.g.*, creatine, methionine, choline) in older women [85]. The accumulation of these metabolites is inversely correlated with the number of oocytes and the proportion of high-quality embryos. This suggests that metabolic dysregulation in the follicular microenvironment is strongly associated with age-related decline in oocyte quality.

##### 4.2.4.5 Microbiome

The microbiome, particularly the gut microbiota, is closely linked to host health. The concept of the “gut-ovary axis” has recently been proposed, suggesting that the microbiome may regulate ovarian aging by affecting hormone levels and inflammation [86].

Santos-Mar Marcos *et al*. found that the gut microbiota structure of premenopausal women differs from that of postmenopausal women and men of the same age [87]. They had a significantly higher ratio of Firmicutes/Bacteroidetes, higher abundance of beneficial butyrate-producing bacteria (*e.g.*, Lachnospira and Roseburia), and lower levels of systemic inflammatory markers. However, after menopause, the composition of the female gut microbiota changes significantly, becoming closer to that of men (*i.e.*, masculinization).

Further research confirms this “masculinization” trend and provides key functional insights [88]. The gut microbiota of premenopausal women is enriched in pathways associated with steroid biosynthesis and degradation compared with those of men and postmenopausal women. These microbial functions are related to host circulating hormones such as testosterone and progesterone, and fecal microbial transplantation experiments support this causal relationship. These findings suggest a close link between ovarian function and gut microbiota, providing a new basis for intervening in ovarian aging from a microbiome perspective.

The integration of multi-omics data is shedding light on the complex genetic and epigenetic networks of ovarian aging, paving the way for personalized reproductive medicine and precision interventions.

In summary, these trends are closely related to the broader field of aging science. Ovarian aging is a “silent catalyst” for systemic age-related diseases [89]. Therefore, advances in ovarian longevity research hold the promise of not only prolonging fertility but also providing interdisciplinary insights for delaying age-related diseases like cardiovascular dysfunction and osteoporosis.

### 4.3 Practical Implications for Clinical Practice and Health Policy

This study provides practical guidance for clinical practice and policy-making. For clinicians, the analysis highlights mitochondrial dysfunction and inflammation as key mechanisms. Therefore, metabolic and anti-inflammatory interventions, such as antioxidants or senolytic agents, may be effective against ovarian aging [40]. For policymakers, ovarian aging should be regarded as a significant health issue rather than merely a natural process. Consistent with recent perspectives [2], we advocate establishing “ovarian aging” as an independent clinical diagnosis. Formally recognizing this diagnosis would help in developing standardized guidelines and provide insurance coverage for preventive care. Ultimately, this approach would improve women's overall health, not just fertility.

### 4.4 Limitations

This study has several limitations. First, the research used data only from the WoSCC database, potentially omitting relevant literature from other databases, such as PubMed and Scopus. However, WoSCC is a globally recognized, high-quality academic research database and is widely used in bibliometric analyses [90]. Our findings should provide a reliable overview of this field. Future bibliometric studies could enhance analysis depth and breadth by using AI-based natural language processing for more precise topic modeling and by developing more robust methods to integrate literature data from multiple databases (*e.g.*, Scopus and PubMed). Second, the cutoff date for this analysis was May 2025. Therefore, the most recent research may not have been fully included. The citation impact of emerging studies has not yet been established, which could lead to underestimating their significance. Finally, this study primarily focuses on natural ovarian aging. While the authors of this study strived to exclude literature on pathological conditions (such as POI), the mechanisms of these diseases partially overlap with those of ovarian aging, which may have affected the precision of the analysis.

## CONCLUSIONS

In conclusion, this study provides a valuable, data-driven landscape of the field. It helps researchers identify established knowledge bases, current research gaps, and promising avenues for future investigation in the field of ovarian aging. Continued exploration in these frontier areas holds the promise of developing novel approaches to delay ovarian aging and preserve female reproductive health.

## Figures and Tables

**Fig. (1):**
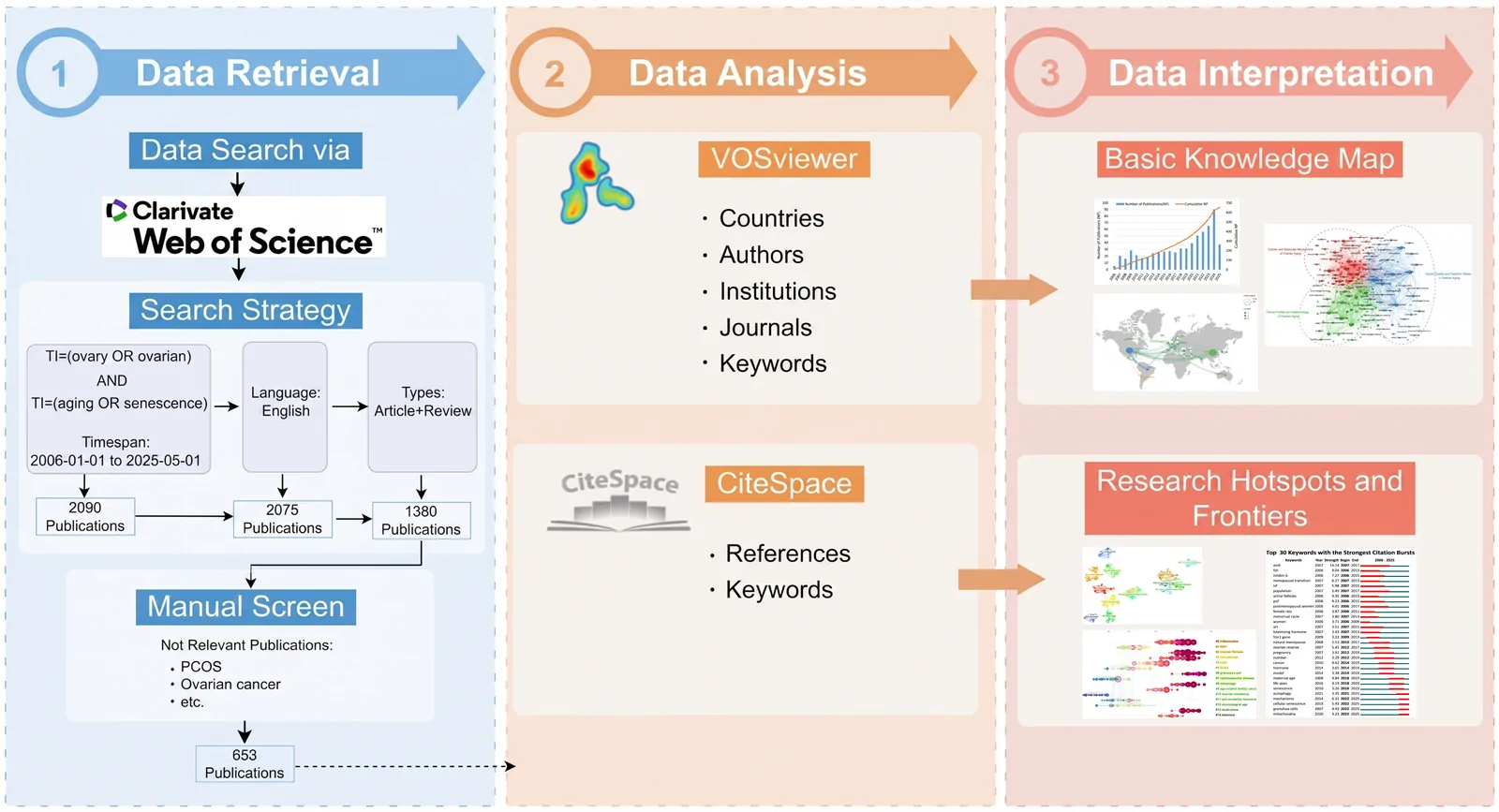
Workflow of the present study.

**Fig. (6):**
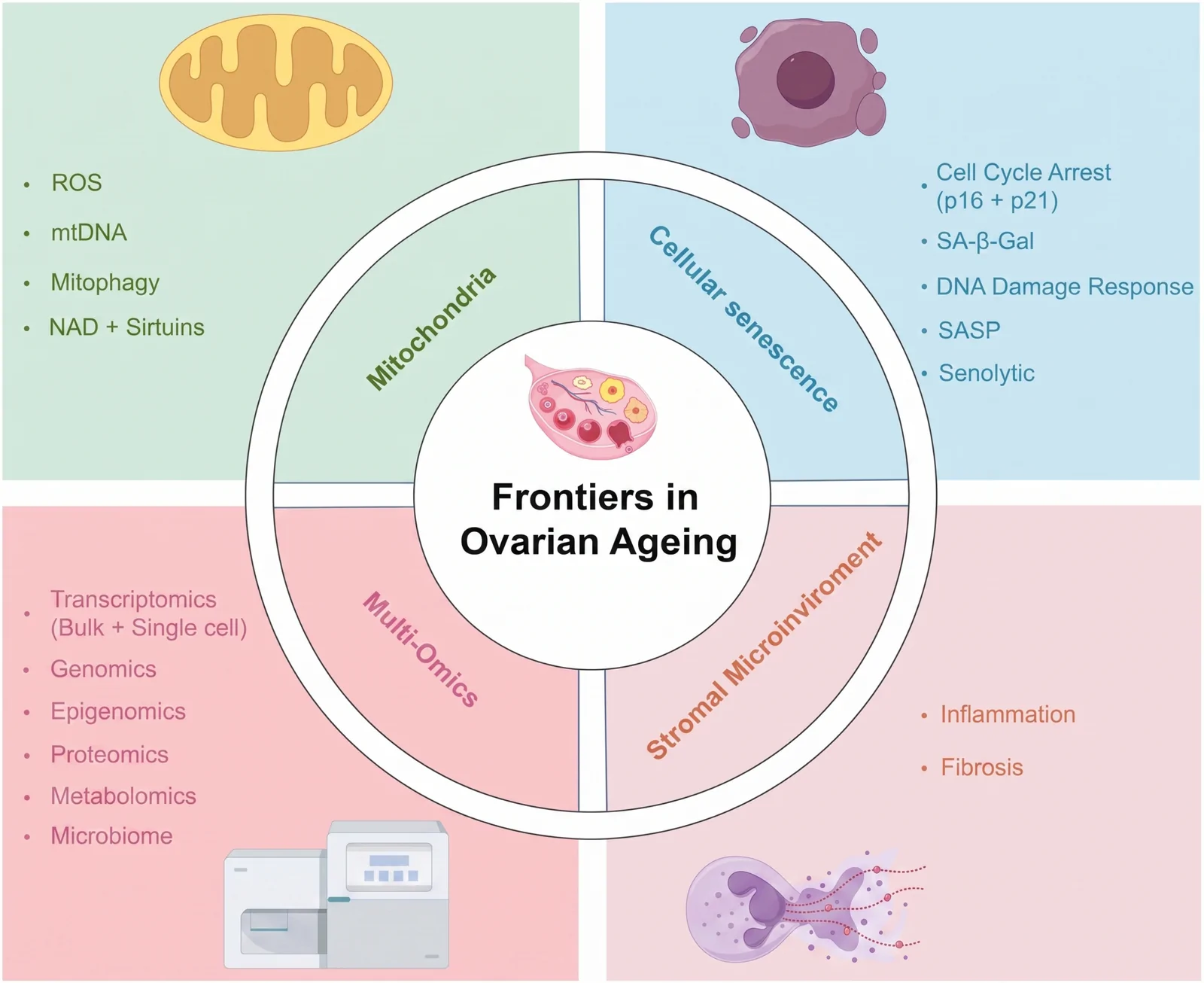
Schematic diagram of the research frontiers in ovarian aging.

**Table 1:** The top 10 most productive countries and institutions in ovarian aging.

Rank	Country	Articles (N)	Percentage (N/653)	Rank	Institution	Articles (N)	Location
1	China	261	39.97%	1	Huazhong Univ Sci & Technol	33	China
2	the USA	199	30.47%	2	Nanjing Med Univ	25	China
3	Italy	29	4.44%	3	Yale Univ	19	the USA
4	Brazil	28	4.29%	4	Zhejiang Univ	17	China
5	Australia	26	3.98%	5	Chinese Acad Sci	14	China
6	the UK	23	3.52%	6	Albert Einstein Coll Med	13	the USA
7	Türkiye	22	3.37%	7	Univ Fed Pelotas	13	Brazil
8	South Korea	21	3.22%	8	Capital Med Univ	11	China
9	Japan	17	2.60%	9	Univ Calif San Francisco	11	the USA
10	Netherlands	14	2.14%	10	Zhengzhou Univ	11	China

**Table 2:** The top 10 most productive and co-cited journals in ovarian aging.

Rank	Productive Journal^a^	Articles (N)	IF	JIF quartile	Rank	Co-cited journal*	Citation (N)	JIF quartile
1	Hum. Reprod.	25	6.1	Q1	1	Hum. Reprod.	1948	Q1
2	Aging-US	24	N/A	N/A	2	Fertil. Steril.	1841	Q1
3	Fertil. Steril.	24	7.0	Q1	3	Biol. Reprod.	1007	Q2
4	Int. J. Mol. Sci.	20	4.9	Q1	4	J. Clin Endocrinol. Metab.	864	Q1
5	J Ovarian Res	20	4.2	Q1	5	Hum. Reprod. Update	780	Q1
6	Menopause-J. N. Am. Menopause Soc.	19	3.0	Q1	6	Reproduction	626	Q1
7	J. Assist. Reprod. Genet.	17	2.7	Q1	7	Endocrinology	612	Q2
8	Front. Endocrinol.	15	4.6	Q1	8	Nature	547	Q1
9	Reprod. Biol. Endocrinol.	15	4.7	Q1	9	Proc. Natl. Acad. Sci. U. S. A.	534	Q1
10	Reprod. Sci.	12	2.5	Q2	10	PLoS One	476	Q2

^a^: All journal titles are ISO abbreviations.

**Table 3:** The top 10 most productive and co-cited authors in ovarian aging.

Rank	Productive Authors	Articles (N)	Percentage (N/653)	Rank	Co-cited Authors	Citation
1	Wang, SX	22	3.37%	1	Broekmans, Fj	208
2	Wu, M	15	2.30%	2	Faddy, Mj	167
3	Schneider, A	14	2.14%	3	Tatone, C	146
4	Zhang, JJ	14	2.14%	4	Gleicher, N	133
5	Gleicher, N	13	1.99%	5	Hansen, Kr	100
6	Luo, AY	11	1.68%	6	Wang, S	98
7	Weghofer, A	11	1.68%	7	La Marca, A	91
8	Barad, DH	10	1.53%	8	May-Panloup, p	86
9	Zhang, Y	10	1.53%	9	Oktay, K	83
10	Masternak, MM	9	1.38%	10	Velde, Ert	83

**Table 4:** The top 10 co-cited references in ovarian aging.

Rank	Co-citation Counts	Author	Title	Type	Journal	Year
1	72	Wang S	Single-Cell Transcriptomic Atlas of Primate Ovarian Aging	Article	Cell	2020
2	35	Park SU	Mechanisms of ovarian aging	Review	Reproduction	2021
3	32	Lliberos C	Evaluation of inflammation and follicle depletion during ovarian ageing in mice	Article	Sci Rep	2021
4	31	Amargant F	Ovarian stiffness increases with age in the mammalian ovary and depends on collagen and hyaluronan matrices.	Article	Aging Cell	2020
5	31	Zhang ZJ	Inflammaging is associated with shifted macrophage ontogeny and polarization in the aging mouse ovary.	Article	Reproduction	2020
6	30	Yang LQ	The Role of Oxidative Stress and Natural Antioxidants in Ovarian Aging	Review	Front Pharmacol	2021
7	26	Yan F	The role of oxidative stress in ovarian aging: a review	Review	J Ovarian Res	2022
8	26	Chiang JL	Mitochondria in Ovarian Aging and Reproductive Longevity	Review	Ageing Res Rev	2020
9	25	Kasapoglu I	Mitochondrial Dysfunction and Ovarian Aging	Review	Endocrinology	2020
10	25	May-Panloup P	Ovarian ageing: the role of mitochondria in oocytes and follicles	Review	Hum Reprod Update	2016

## Data Availability

The datasets used and/or analysed during the current study are available from the corresponding author upon reasonable request.

## LIST OF ABBREVIATIONS

POI: Premature Ovarian Insufficiency
WoSCC: Web of Science Core Collection
ROS: Reactive Oxygen Species
mtDNA: Mitochondrial DNA
NAD+: Nicotinamide Adenine Dinucleotide
DDR: DNA Damage Response
SA-β-Gal: Senescence-Associated β-Galactosidase
SASP: Senescence-Associated Secretory Phenotype
MMP13: Matrix Metalloproteinase 13
Bulk RNA-seq: Bulk RNA-sequencing
scRNA-seq: Single-cell RNA-sequencing
GWAS: Genome-Wide Association Studies
ANM: Age at Natural Menopause
rDNA: Ribosomal DNA
LLPs: Long-Lived Proteins

## SUPPLEMENTARY MATERIAL

Supplementary material is available on the publisher's website along with the published article.
**Supplementary Table S1** **:** List of Publications with Co-Citation Frequencies ≥10 in Ovarian Aging
**Supplementary Table S2** **:** Top 25 References with the Strongest Citation Bursts in Ovarian Aging
