# Supplementary material for: Research Hotspots and Emerging Frontiers in Ovarian Aging: A Bibliometric Analysis (2006–2025)
Source: Endocr Metab Immune Disord Drug Targets. 2026 Apr 28;26:E18715303441084. doi: 10.2174/0118715303441084260119050045 (PMC13595331; doi:10.2174/0118715303441084260119050045)
Supplement: Supplementary file 1 [file EMIDDT-26-E18715303441084_SD1.pdf]

Supplementary Material

Research Hotspots and Emerging Frontiers in Ovarian Aging: A Bibliometric Analysis (2006–2025)

Qingquan Gong[1,2,#], Wenhong Ma[1,3,#], Chao Yang[1,2], Dingyu Liang[1,2], Yifu Wang[1,2], Yihua Yang[1,2,*] and Mingyou Dong[1,4,*]

[1]Guangxi Reproductive Medical Center, the First Affiliated Hospital of Guangxi Medical University, Nanning, 530021 China; [2]Key Laboratory of Early Prevention and Treatment for Regional High Frequency Tumor (Guangxi Medical University), Ministry of Education, Nanning, 530021, China; [3]Reproductive Medicine Center, Nanning Women and Children's Hospital, Nanning, 530011, China; [4]Guangxi Engineering Research Center for Precise Genetic Testing of Long-dwelling Nationalities, Youjiang Medical University for Nationalities, Baise, 533000, China

Supplementary Table S1: List of publications with co-citation frequencies >= 10 in ovarian aging.

Rank	Co-citation counts	Year	Author	Journal	DOI
1	72	2020	Wang S	CELL	DOI 10.1016/j.cell.2020.01.009
2	35	2021	Park SU	REPRODUCTION	DOI 10.1530/REP-21-0022
3	32	2021	Lliberos C	SCI REP-UK	DOI 10.1038/s41598-020-79488-4
4	31	2020	Amargant F	AGING CELL	DOI 10.1111/acel.13259
5	31	2020	Zhang ZJ	REPRODUCTION	DOI 10.1530/REP-19-0330
6	30	2021	Yang LQ	FRONT PHARMA-COL	DOI 10.3389/fphar.2020.617843
7	26	2022	Yan F	J OVARIAN RES	DOI 10.1186/s13048-022-01032-x
8	26	2020	Chiang JL	AGEING RES REV	DOI 10.1016/j.arr.2020.101168
9	25	2020	Kasapoglu I	ENDOCRINOLOGY	DOI 10.1210/endocr/bqaa001
10	25	2016	May-Panloup P	HUM REPROD UP-DATE	DOI 10.1093/humupd/dmw028
11	24	2021	Tesarik J	INT J MOL SCI	DOI 10.3390/ijms22031371
12	24	2019	Zhang JJ	AGING-US	DOI 10.18632/aging.101784
13	23	2022	Umehara T	SCI ADV	DOI 10.1126/sciadv.abn4564
14	23	2020	Bertoldo MJ	CELL REP	DOI 10.1016/j.celrep.2020.01.058
15	22	2019	Sasaki H	FRONT ENDO-CRINOL	DOI 10.3389/fendo.2019.00811
16	21	2019	Huang YQ	J IMMUNOL RES	DOI 10.1155/2019/8069898
17	20	2022	Secomandi L	HUM REPROD UP-DATE	DOI 10.1093/humupd/dmab038

18	20	2016	Briley SM	REPRODUCTION	DOI 10.1530/REP-16-0129
19	19	2021	Wang L	J CELL PHYSIOL	DOI 10.1002/jcp.30468
20	19	2021	Ruth KS	NATURE	DOI 10.1038/s41586-021-03779-7
21	19	2021	Ansere VA	MECH AGEING DEV	DOI 10.1016/j.mad.2020.111425
22	18	2021	Foley KG	REPRODUCTION	DOI 10.1530/REP-20-0489
23	18	2017	Tamura H	J PINEAL RES	DOI 10.1111/jpi.12381
24	18	2009	Broekmans FJ	ENDOCR REV	DOI 10.1210/er.2009-0006
25	17	2017	Dou XW	AGING CELL	DOI 10.1111/acel.12617
26	17	2008	Hansen KR	HUM REPROD	DOI 10.1093/humrep/dem408
27	16	2021	Chon SJ	FRONT CELL DEV BIOL	DOI 10.3389/fcell.2021.672890
28	16	2021	Li CJ	AGING DIS	DOI 10.14336/AD.2020.1113
29	16	2020	Mara JN	AGING-US	DOI 10.18632/aging.103237
30	16	2011	Broer SL	J CLIN ENDOCR METAB	DOI 10.1210/jc.2010-2776
31	15	2021	Lliberos C	FRONT CELL DEV BIOL	DOI 10.3389/fcell.2020.628473
32	15	2020	Miao YL	CELL REP	DOI 10.1016/j.celrep.2020.107987
33	15	2019	Laisk T	HUM REPROD UP-DATE	DOI 10.1093/humupd/dmy031
34	14	2024	Isola JVV	NATURE AGING	DOI 10.1038/s43587-023-00552-5
35	14	2022	Wu JC	FRONT ENDO-CRINOL	DOI 10.3389/fendo.2022.952471
36	14	2021	Navarro-Pando JM	SCI ADV	DOI 10.1126/sciadv.abc7409
37	14	2020	Turan V	HUM REPROD UP-DATE	DOI 10.1093/humupd/dmz043
38	14	2020	Wagner M	NAT COMMUN	DOI 10.1038/s41467-020-14936-3
39	14	2011	Hansen KR	FERTIL STERIL	DOI 10.1016/j.fertnstert.2010.04.006
40	14	2008	Sowers MR	J CLIN ENDOCR METAB	DOI 10.1210/jc.2008-0567
41	13	2022	Landry DA	SCI ADV	DOI 10.1126/sciadv.abq1475
42	13	2022	Camaioni A	J ASSIST REPROD GEN	DOI 10.1007/s10815-022-02478-0
43	13	2020	Kinnear HM	REPRODUCTION	DOI 10.1530/REP-19-0501

44	13	2010	Wallace WHB	PLOS ONE	DOI 10.1371/journal.pone.0008772
45	12	2023	Zhang Y	NATURE AGING	DOI 10.1038/s43587-023-00498-8
46	12	2020	Yang QL	FREE RADICAL BIO MED	DOI 10.1016/j.freeradbiomed.2020.05.003
47	12	2019	Duffy DM	ENDOCR REV	DOI 10.1210/er.2018-00075
48	12	2019	Fan X	NAT COMMUN	DOI 10.1038/s41467-019-11036-9
49	12	2018	Liu XT	OXID MED CELL LONGEV	DOI 10.1155/2018/9390810
50	11	2023	López-Otin C	CELL	DOI 10.1016/j.cell.2022.11.001
51	11	2023	Ben Yaakov T	ELIFE	DOI 10.7554/eLife.74915
52	11	2020	Mikwar M	MUTAT RES-REV MUTAT	DOI 10.1016/j.mrrev.2020.108320
53	11	2020	McCloskey CW	CLIN CANCER RES	DOI 10.1158/1078-0432.CCR-19-0603
54	11	2018	Zhang YY	MOL CELL	DOI 10.1016/j.molcel.2018.10.029
55	11	2013	Dólleman M	J CLIN ENDOCR METAB	DOI 10.1210/jc.2012-3995
56	11	2012	Freeman EW	J CLIN ENDOCR METAB	DOI 10.1210/jc.2011-3032
57	10	2023	Cavalcante MB	GEROSCIENCE	DOI 10.1007/s11357-023-00768-8
58	10	2021	van der Reest J	AGEING RES REV	DOI 10.1016/j.arr.2021.101378
59	10	2020	Peters AE	ANTIOXID REDOX SIGN	DOI 10.1089/ars.2019.7986
60	10	2019	Spears N	HUM REPROD UP-DATE	DOI 10.1093/humupd/dmz027
61	10	2019	Garcia DN	GEROSCIENCE	DOI 10.1007/s11357-019-00087-x
62	10	2017	Wang TR	AM J REPROD IM-MUNOL	DOI 10.1111/aji.12651
63	10	2011	Kelsey TW	PLOS ONE	DOI 10.1371/journal.pone.0022024

Supplementary Table S2: Top 25 references with the strongest citation bursts in ovarian aging.

References	Year	Strength	Begin	End	2006 - 2025
Hansen KR, 2008, HUM REPROD, V23, P699, DOI 10.1093/humrep/dem408, DOI	2008	9.05	**2008**	2013	
Sowers MR, 2008, J CLIN ENDOCR METAB, V93, P3478, DOI 10.1210/jc.2008-0567, DOI	2008	7.44	**2008**	2013	
Broekmans FJ, 2009, ENDOCR REV, V30, P465, DOI 10.1210/er.2009-0006, DOI	2009	10.09	**2010**	2015	
Broer SL, 2011, J CLIN ENDOCR METAB, V96, P2532, DOI 10.1210/jc.2010-2776, DOI	2011	8.37	**2012**	2017	
Hansen KR, 2011, FERTIL STERIL, V95, P170, DOI 10.1016/j.fertnstert.2010.04.006, DOI	2011	7.32	**2012**	2017	
Wallace WHB, 2010, PLOS ONE, V5, P0, DOI 10.1371/journal.pone.0008772, DOI	2010	7.27	**2012**	2015	
Freeman EW, 2012, J CLIN ENDOCR METAB, V97, P1673, DOI 10.1210/jc.2011-3032, DOI	2012	5.82	**2012**	2017	
Kelsey TW, 2011, PLOS ONE, V6, P0, DOI 10.1371/journal.pone.0022024, DOI	2011	5.55	**2012**	2017	
Dólleman M, 2013, J CLIN ENDOCR METAB, V98, P2106, DOI 10.1210/jc.2012-3995, DOI	2013	6.35	**2014**	2017	
May-Panloup P, 2016, HUM REPROD UPDATE, V22, P725, DOI 10.1093/humupd/dmw028, DOI	2016	12.25	**2016**	2021	
Briley SM, 2016, REPRODUCTION, V152, P245, DOI 10.1530/REP-16-0129, DOI	2016	10.3	**2018**	2021	
Tamura H, 2017, J PINEAL RES, V62, P0, DOI 10.1111/jpi.12381, DOI	2017	7.57	**2018**	2023	
Dou XW, 2017, AGING CELL, V16, P825, DOI 10.1111/acel.12617, DOI	2017	7.15	**2018**	2023	
Wang TR, 2017, AM J REPROD IMMUNOL, V77, P0, DOI 10.1111/aji.12651, DOI	2017	5.66	**2020**	2021	
Zhang ZJ, 2020, REPRODUCTION, V159, P325, DOI 10.1530/REP-19-0330, DOI	2020	5.59	**2020**	2023	
Song C, 2016, SCI REP-UK, V6, P0, DOI 10.1038/srep35165, DOI	2016	5.09	**2020**	2021	
Yan F, 2022, J OVARIAN RES, V15, P0, DOI 10.1186/s13048-022-01032-x, DOI	2022	7.67	**2022**	2025	
Park SU, 2021, REPRODUCTION, V162, PR19, DOI 10.1530/REP-21-0022, DOI	2021	7.54	**2022**	2025	
Umehara T, 2022, SCI ADV, V8, P0, DOI 10.1126/sciadv.abn4564, DOI	2022	6.77	**2022**	2025	
Wang S, 2020, CELL, V180, P585, DOI 10.1016/j.cell.2020.01.009, DOI	2020	6.61	**2022**	2025	
Yang LQ, 2021, FRONT PHARMACOL, V11, P0, DOI 10.3389/fphar.2020.617843, DOI	2021	6.46	**2022**	2025	
Lliberos C, 2021, SCI REP-UK, V11, P0, DOI 10.1038/s41598-020-79488-4, DOI	2021	6.44	**2022**	2025	
Wang L, 2021, J CELL PHYSIOL, V236, P7966, DOI 10.1002/jcp.30468, DOI	2021	6.04	**2022**	2025	
Chiang JL, 2020, AGEING RES REV, V63, P0, DOI 10.1016/j.arr.2020.101168, DOI	2020	5.22	**2022**	2025	
Secomandi L, 2022, HUM REPROD UPDATE, V28, P172, DOI 10.1093/humupd/dmab038, DOI	2022	5.18	**2022**	2025	
